# Development of Composite Scaffolds Based on Cerium Doped-Hydroxyapatite and Natural Gums—Biological and Mechanical Properties

**DOI:** 10.3390/ma12152389

**Published:** 2019-07-26

**Authors:** Marcus Vinicius Beserra dos Santos, Lorenna Bastos Nogueira Rocha, Ewerton Gomes Vieira, Ana Leite Oliveira, Anderson Oliveira Lobo, Maria Acelina Martins de Carvalho, Josy Anteveli Osajima, Edson Cavalcanti Silva-Filho

**Affiliations:** 1 LIMAV, Interdisciplinary Laboratory for Advanced Materials, Federal University of Piaui, Campus Universitário Ministro Petrônio Portella, Teresina, 64049-550 Piaui, Brazil; 2 NUPCELT, Interdisciplinary Laboratory for Advanced Materials, Federal University of Piaui, Campus Universitário Ministro Petrônio Portella, Teresina, 64064-260 Piaui, Brazil; 3 Center of Biotechnology and Fine Chemical, Universidade Catolica Portuguesa, 4169-005 Porto, Portugal

**Keywords:** calcium phosphate, doping, scaffold

## Abstract

Hydroxyapatite (HAp) is a ceramic material composing the inorganic portion of bones. Ionic substitutions enhance characteristics of HAp, for example, calcium ions (Ca^2+^) by cerium ions (Ce^3+^). The use of HAp is potentialized through biopolymers, cashew gum (CG), and gellan gum (GG), since CG/GG is structuring agents in the modeling of structured biocomposites, scaffolds. Ce-HApCG biocomposite was synthesized using a chemical precipitation method. The obtained material was frozen (–20 °C for 24 h), and then vacuum dried for 24 h. The Ce-HApCG was characterized by X-Ray diffractograms (XRD), X-ray photoemission spectra (XPS), Fourier transform infrared spectroscopy (FTIR), field emission scanning electron microscopy (FESEM), and energy dispersive spectroscopy (EDS). XRD and FTIR showed that Ce-HApCG was successfully synthesized. XRD showed characteristic peaks at 2θ = 25.87 and 32.05, corresponding to the crystalline planes (0 0 2) and (2 1 1), respectively, while phosphate bands were present at 1050 cm^−1^ and 1098 cm^−1^, indicating the success of composite synthesis. FESEM showed pores and incorporated nanostructured granules of Ce-HApCG. The mechanical test identified that Ce-HApCG has a compressive strength similar to the cancellous bone’s strength and some allografts used in surgical procedures. In vitro tests (MTT assay and hemolysis) showed that scaffold was non-toxic and exhibited low hemolytic activity. Thus, the Ce-HApCG has potential for application in bone tissue engineering.

## 1. Introduction

Hydroxyapatite (HAp), Ca_10_(PO_4_)_6_(OH)_2_, is a bioceramic that is widely studied today. The motivational factor is based on the compatibility and chemical similarity between hydroxyapatite and various parts of the human body, including bone and dental tissues [1]. Albbe began the studies of these materials in 1920 with the use of tricalcium phosphate. However, it was only in 1974 that Levitt [2] and Monroe et al. [3] published the application of this phosphate in dentistry. Currently, among the motivating factors of interest in these biomaterials is the fact that calcium phosphates are osteogenic, osteoconductive, and osteoinductive materials [4,5,6,7], added to excellent results when used as materials for orthopedic implant coatings and as substitute materials for parts of the human body [1,4,8]. However, pure HAp may present low reabsorption by the organism [9], which affects its bioactivity. For example, the low toughness that this bioceramic presents makes the HAp easily fracture in the face of possible efforts [4,10,11].

Faced with these challenges, ionic substitutions are shown as an alternative to improve the mechanical and biological properties of HAp. Calcium ions (Ca^2+^) present in the structure of HAp can be replaced by divalent and trivalent ions, such as Sr^2+^, Mg^2+^, Zn^2+^, Ce^3+^, La^3+^, and so on [9,12,13,14,15,16]. Cerium ions (Ce^3+^) have been widely used as an enhancement agent for the chemical and biological properties of some materials. For example, drugs containing cerium are used because of the need for a low concentration of the ions that add up to the long duration of the biocidal action. Biocompatibility and reabsorption are other characteristics that the incorporation of cerium provides to materials [17,18,19].

Cerium can also act in a manner similar to calcium in the human organism, because it accumulates in organisms in small quantities, thus providing biocompatibility and reabsorption of the material in the tissue region [20,21]. Periodic trends such as electronegativity and ionic radius (1.01 and 0.100 nm to Ca^2+^, and 1.06 and 0.107 nm to Ce^3+^, respectively) are quite similar for both ions [17]. This similarity of properties allows an ionic substitution of the present calcium of the HAp structure by cerium [22].

The development of new composites has been through the use of various biopolymers, which due to their natural origin, are abundant in nature, non-toxic, and add characteristics such as biodegradability and biocompatibility to new materials [23,24]. Among the biopolymers, cashew gum (CG) has gained prominence in recent years. CG is a polysaccharide composed of galactose (72%), D-glucose (14%), glucuronic acid (4.7%), arabinose (4.6%), and rhamnose (3.2%) [25,26,27].

Among the properties of the biopolymers already mentioned, CG may act as the coating agent, in addition to possessing property of anti-inflammatory agent, healing agent [28], antimicrobial [29], binding agent or adhesive, and so on [30,31]. CG is obtained from the bark of the cashew tree (*Anacardium occidentale L.*, family: *Anacardiaceae*) and is produced in the epithelial cells of the plant (exudate) [31,32,33]. Currently, *Anacardium occidentale L.* has about 1.12 million hectares planted around the world, with Brazil and India accounting for 91% of the commercial exports of products obtained from *Anacardium occidentale L.* [34,35].

The composition of scaffold is completed with the use of gellan gum (GG), a biopolymer that has gained prominence for its application in the pharmaceutical and food industry. The insertion into the scaffold formulation of GG is essential, since the gum has a unique characteristic in forming a gel when in aqueous solution or when it is in the presence of metallic ions [36].

GG is an extracellular anionic polysaccharide which is a product of the excretion of bacteria of the type *Sphingonomas elodea* (ATCC 31,461), produced by aerobic fermentation. GG is constituted from monosaccharides of repeating units with a linear chemical structure consisting of 1,3β-D-glucose, 1,4β-D-glucuronic acid, 1,4β-D-glucose, and 1,4α-rhamnose [36,37,38,39].

In this way, the present research aims to synthesize and characterize scaffold based on cerium-doped hydroxyapatite, cashew gum, and gellan gum for biomedical applications in bone grafts. The evaluation of its mechanical and biological properties by the MTT and hemolysis assays results are carried on to enhance the in vivo assays, hoping to obtain a material that can replace small bone parts that are already in the body. The material provides anti-inflammatory action, and healing action added to the antibacterial action.

## 2. Results and Discussion

### 2.1. Characterizations

**Figure 1:**
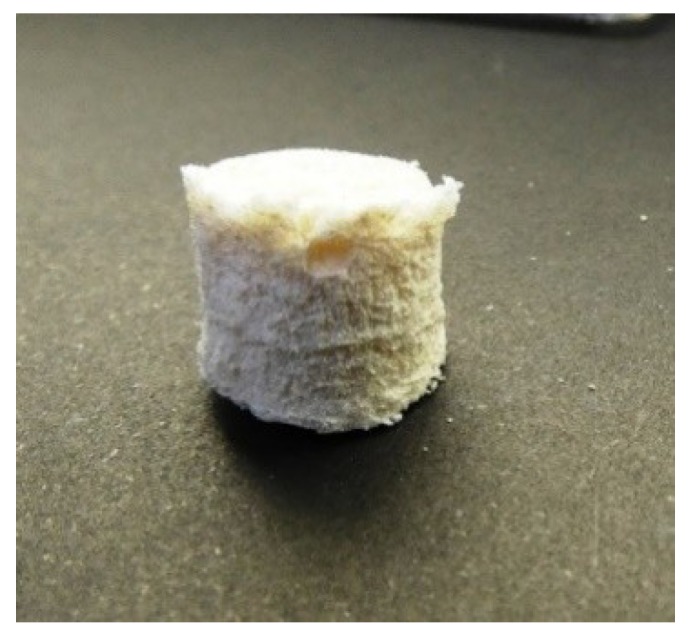
Scaffold obtained in this study.

The scaffold was obtained by means of a cylindrical mold with physical characteristics corresponding to the mold used in the synthesis with a slightly yellowish coloration and a cylindrical area of 13.48 mm in diameter and 13.54 mm in height. The scaffolds were homogeneous in the macroscopic level, without cracks, presenting a spongy aspect with a certain degree of tension and compression. Figure 1 shows the image of the scaffold obtained.

**Figure 2:**
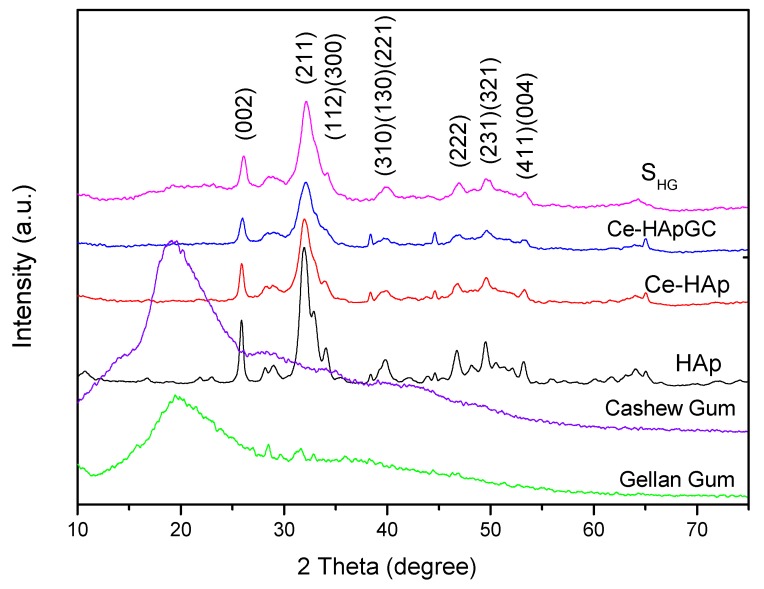
X-Ray diffractograms (XRD) patterns of powders hydroxyapatite (HAp), cerium (Ce)-HAp, Ce-HAp cashew gum (CG), the scaffold based on Ce-HApCG+gellan gum (GG) (S*_HG_*), cashew gum, and gellan gum.

Figure 2 shows the X-Ray diffractograms for HAp, Ce-HAp, Ce-HApCG, the scaffold based on Ce-HApCG+GG (S*_HG_*), cashew gum, and gellan gum. The obtained diffractograms for the HAp and Ce-HAp were compared with the database available in the X’Pert HighScorePlus software and all peaks were indexed with JCPDS data (JCPDS Card number: 00-003-0747). Finally, it was also observed that Ce-HAp showed no changes in its lattice parameter in relation to pure hydroxyapatite The comparison between the XRD patterns show the presence of the plans (0 0 2), (2 1 1), (1 1 2), (3 0 0), (3 1 0), (1 3 0), (2 2 1), (2 2 2), (2 3 1), (3 2 1), (4 1 1) and (0 0 4) [9,40,41,42,43,44].

The gum’s diffractograms were quite similar, with a broad diffused peak at 2*θ* = 20°. This characteristic is commonly observed in amorphous materials, meaning, this result indicates a low degree of crystallinity and low structural organization, commonly attributed to polymers.

**Figure 3:**
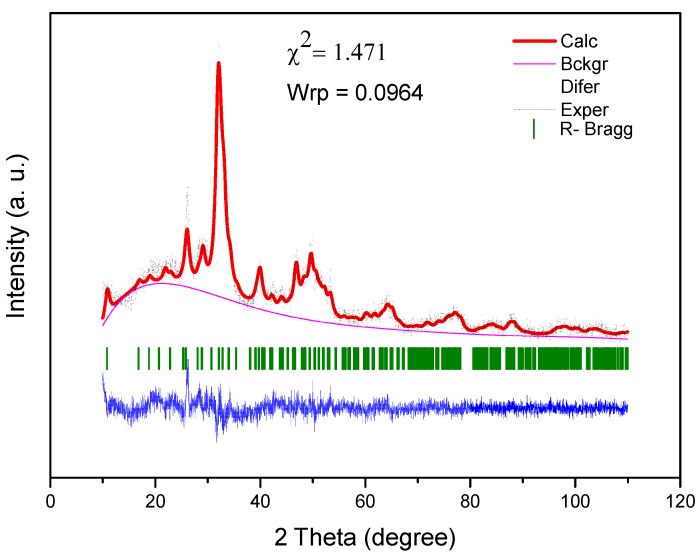
Rietveld refinement of S*_HG_* scaffold.

An important information is that the diffractogram shown refers to the presence of CG and GG in the scaffold composition, since the S*_HG_* diffractogram showed no changes in the peaks of the doped-HAp, as can be observed when the diffractograms of Ce-HApCG and S*_HG_* are compared. In fact, it is observed that there is only a subtle alteration in the baseline, especially in the region between 15 to 30° in which the main information of the gums in the diffractogram appears. The modification at the baseline is shown in Figure 3. The S*_HG_* diffractogram confirms the continuity of the crystalline and organized structure [43,44].

The Rietveld method was performed for the diffractogram of the S*_HG_* sample. The Rietveld refinement, performed in the EXPGUI/GSAS software, revealed the presence of a hexagonal crystal system with a P63/m space group and *Laue group 6/m*, with the following lattice parameters: A = b = 9.447481, and c = 6.897580 Å, and unit cell volume of 533.162328 Å^3^ [45]. Figure 3 shows the result of the Rietveld refinement. Rietveld refinement is qualified as satisfactory as to the empirically obtained value of χ^2^ and values equal 2.00 or less are satisfactory. The obtained χ^2^ value was relatively low: 1.471.

**Figure 4:**
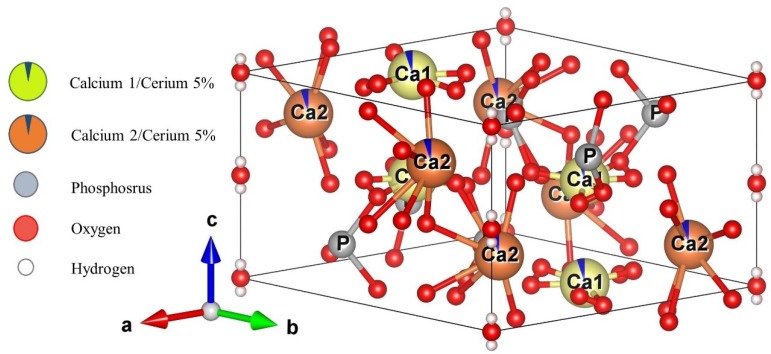
Simulated representation of the Ce-Hap unit cell of that composes S*_HG_*.

Figure 4 shows a schematic representative of the Ce-HAp unit cell synthesized in this research. The unit cell was simulated using the VESTA (Visualization for Electronic and Structural Analysis) software [46] and also using the lattice parameter that were obtained by Rietveld method. The simulation performed in the software did not allow the inclusion of parts of the substituent ion, so the doping ion, i.e., the cerium, is represented by dark blue parts present in the Ca1 and Ca2 atoms.

**Figure 5:**
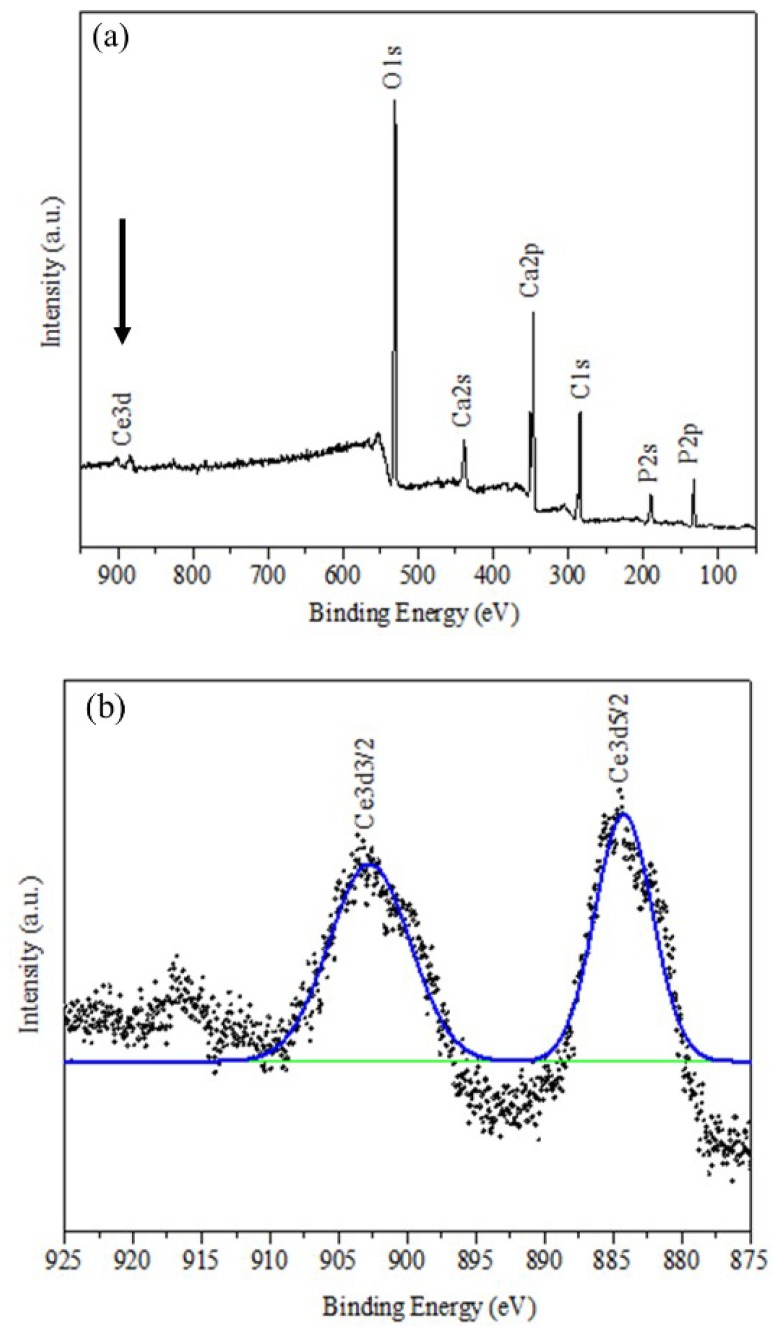
XPS spectrum for (**a**) Ce-HAp and (**b**) binding energy (B.E) corresponding to the 3d5/2 and 3d3/2 pairs of spin-orbit doublets.

The presence of cerium in Ce-HAp was confirmed by XPS. Thus, the elements that constitute Ce-HAp were detected: Peaks at 440 eV (Ca2s), 348 eV (Ca2p), 188 eV (P2s), 132 eV (P2p), and 530 eV (O1s). It was also possible to detect discrete emission lines (875–925 eV) related to the electron doublets ejected from the Ce 3d orbital (Figure 5b). The electronic state assigned to position v and u revealed excitations in binding energy (B.E) corresponding to the 3d5/2 and 3d3/2 pairs of spin-orbit doublets. This type of spin-orbit coupling corroborates with existence of the cerium in the structure of Ce-HAp [47].

**Figure 6:**
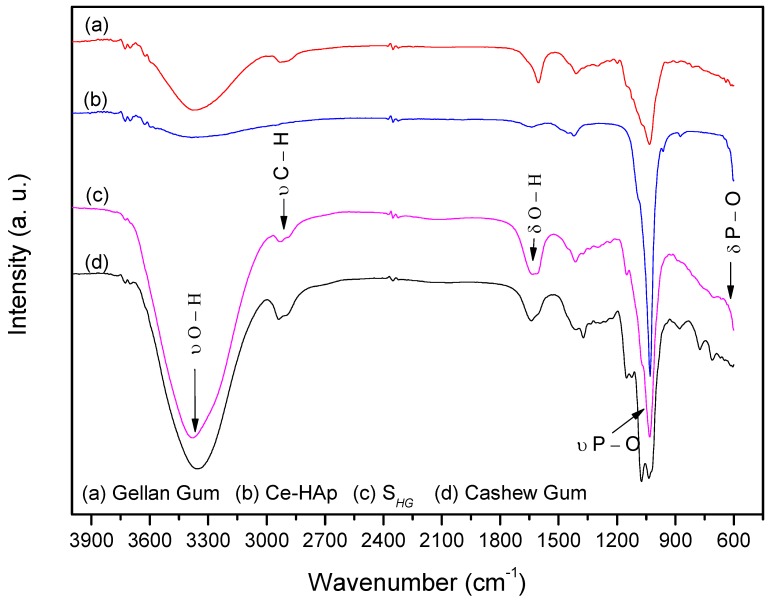
FTIR spectra of scaffold and precursor materials: (a) Gellan gum, (b) Ce-Hap, (c) S*_HG_*, and (d) cashew gum.

FTIR spectra of the precursor materials used in the synthesis of scaffold and S*_HG_* material are shown in Figure 6. When analyzing the spectrum of Ce-HAp, it is possible to observe a band at 3400 cm^−1^ corresponding to the stretching of OH groups from the Ce-doped hydroxyapatite structure, as well as the hydroxyl groups of water molecules adsorbed on the surface of the material. Another band is shown at 1649 cm^−1^, this band corresponds to a deformation also of OH groups. In the Ce-HAp spectrum, the asymmetric deformation of the phosphate groups (PO_4_^3−^) is observed in the region at 1098 cm^−1^ and 1050 cm^−1^. The presence of phosphate groups is also shown at 611 cm^−1^. This band is related to P–O asymmetric deformation. Finally, the P–O (H) group presents a band at 566 cm^−1^ referring to the asymmetric deformation of the HPO_4_^2−^ group that chemically compose the hydroxyapatite structure [48].

In the CG spectrum, the band at 3300 cm^−1^ attributing to the stretching of OH groups was observed. A band at 2920 cm^−1^ refers to the stretching of C–H groups from the carbonic chains of the monomers that chemically constitute the CG. The band at 1649 cm^−1^ refers to deformation vibrations of OH groups [49] and bands at 1150, 1074 and 1035 cm^−1^ refer to glycosidic bonds of type C–O–C [48,49,50].

The spectrum of the GG showed stretching of the OH group in the region at 3300 cm^−1^. In the region at 2920 cm^−1^ a peak was observed for the stretching of C–H groups. The band for the drawing of COO^−^ groups was observed at 1592 cm^−1^ and a peak at 1035 cm^−1^ corresponds to the stretching of the C–O–C group of the glycosidic bonds pertaining to the biopolymer [51,52].

By analyzing the scaffold spectrum, it was possible to perceive characteristics of the precursor materials in the material composition. The band at 3400 cm^−1^ refers to a stretching vibration of OH groups present in the chemical composition of Ce-HAp and the monomers constituting the CG and GG. A band at 1649 cm^−1^ was also observed, resulting from a deformation of the OH groups. The bands that appeared in the spectrum of S*_HG_* in the region at 1098 cm^−1^ and 1051 cm^−1^ refers to the asymmetric deformation of the phosphate (PO_4_^3−^) groups. The phosphate groups also appeared at 611 cm^−1^. These bands are related to the asymmetric deformation of the P–O type. Such bands are characteristic of Ce-HAp [48]. The discrete band at 2920 cm^−1^ refers to the C–H groups of carbonic chains of the monomers constituting the CG and GG.

The band on the scaffold spectrum at 1149 cm^−1^ is derived from the C–O type bonds present in the monomers of both gums, as well as the bands present at 1627 and 1409 cm^−1^, which are related to the COO^–^ group [48,49,50,51,52], and finally, in the region at 1035 cm^−1^ was observed presence of the band referring to the glycosidic bonds present in both gums [48,49,50,51,52].

**Figure 7:**
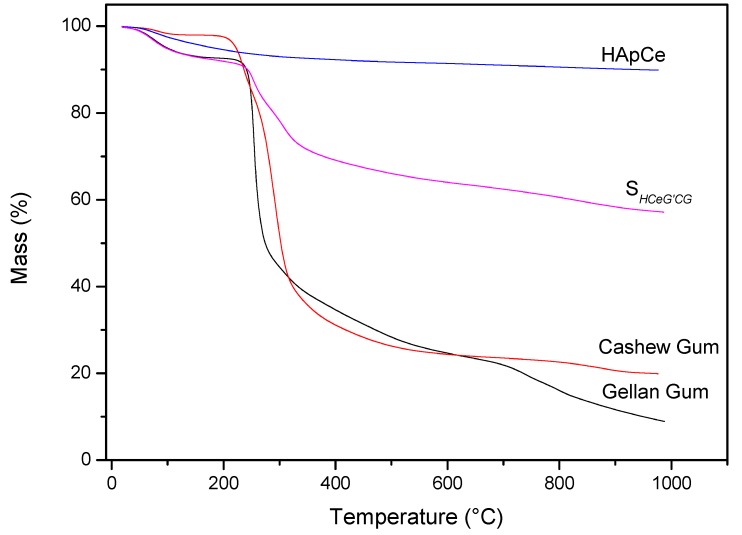
TG curves of precursors materials and S*_HG_*.

Analysis of the materials in relation to thermal stability occurred by TG/DTG. Figure 7 shows TG curves of the precursor materials and the scaffold. Analyzing the thermogravimetric curve of the CG, it was observed the occurrence of two events. The first thermal event occurred at approximately 35 °C, ending at 129 °C. This event is caused by the loss of water in the sample, resulting in about 1.78% mass loss of the CG. The second thermal event occurred at 286 °C. This event is related to the breakdown of the polysaccharide structure, resulting in its decomposition, besides the decomposition of some residues present in the gums, with 73.05% mass loss [53]. In the region between 29 to 130 °C, the first mass loss of S*_HG_* was found. This loss can be attributed to water found adsorbed on the scaffold, with 6.15% mass loss.

For the TG curve of the Ce-doped hydroxyapatite, only one thermal event related to mass loss was observed at a temperature of approximately 100 °C. This loss of mass can be attributed to the exit of water adsorbed on the surface of Ce-HAp. The value of mass loss in this event was 2.7% [54,55]. The TG analysis of the GG showed two thermal events with the first event occurring in the range of 27 to 124 °C, corresponding to 5.87% of the mass loss attributed to water [56,57]. The second thermal event occurs between 195 to 353 °C, referring to the degradation of the polymer, with the mass variation to 54.54% [56,57]. The TG curve of the S*_HG_* showed that the scaffold presented the same thermal events of the precursors materials. When comparing the value, in percentage of mass of the Ce-HAp, an increase in the value of the mass was noticed. The addition is attributed to the sum of the water mass adsorbed on the hydroxyapatite to the mass of the two gums present in the scaffold structure. The second thermal event appeared in the range of 200 to 572 °C with a mass loss of 27.44%. This loss of mass is attributed to the decomposition of the polysaccharide structure of CG and GG present in scaffold [53,56,57].

**Figure 8:**
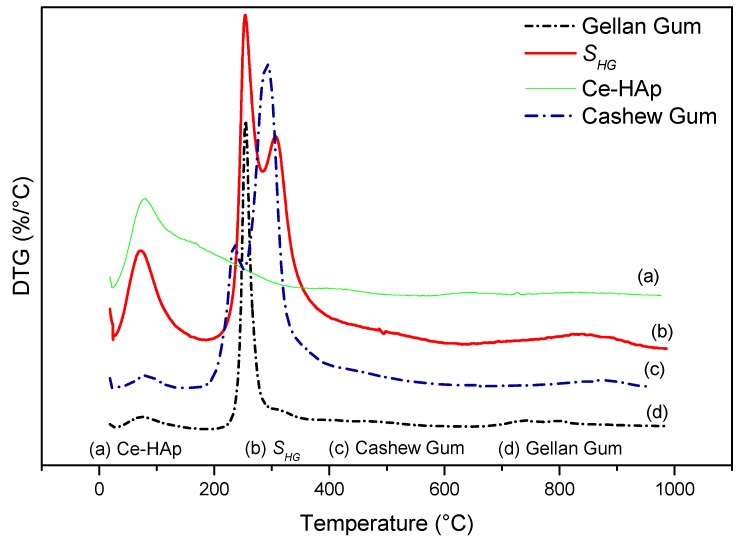
DTG of (a) Ce-HAp, (b) S*_HG_*, (c) cashew gum, and (d) gellan gum.

Figure 8 shows the DTG curves of cashew gum, Ce-HAp, S*_HG_*, and gellan gum. By means of the data obtained from the DTG curves it was possible to elucidate the number of thermal events mentioned and discussed previously. From the thermal degradation curve of the scaffold, a probable characteristic of the material was observed. In other words, it was noted that there may be a possibility of sterilizing the material in an autoclave, since the thermal event that occurred around 120 °C refers to the loss of water mass. This fact is cited in this study because the scaffold is a material that can be applied in the biomedical area, and this information is highlighted by the fact that sterilization via an autoclave is one of the most common methods practiced in biomedical laboratories [58].

**Figure 9:**
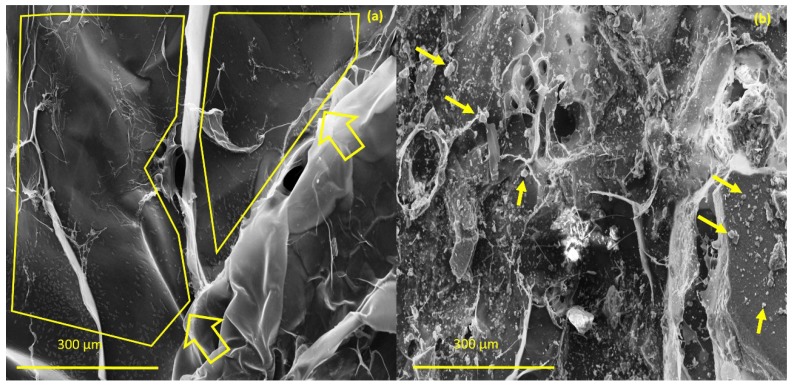
(**a**) Field emission scanning electron microscopy (FESEM) of pure gellan gum (**b**) and FESEM of scaffold.

The investigation morphology and porosity of the scaffolds was performed by FESEM. Figure 9 shows a smooth and translucent surface with aggregates of Ce-HAp particles distributed along the material as well as in the inner layers of the scaffold. The smooth layer is attributed to the polymer network of GG, this assertion is proven when comparing with the micrograph of GG (Figure 9a). Figure 9b shows white-colored agromellated microparticles, attributed to hydroxyapatite that is dispersed over the polymer network. It is also possible to notice a distribution of Ce-HApCG microparticles.

**Figure 10:**
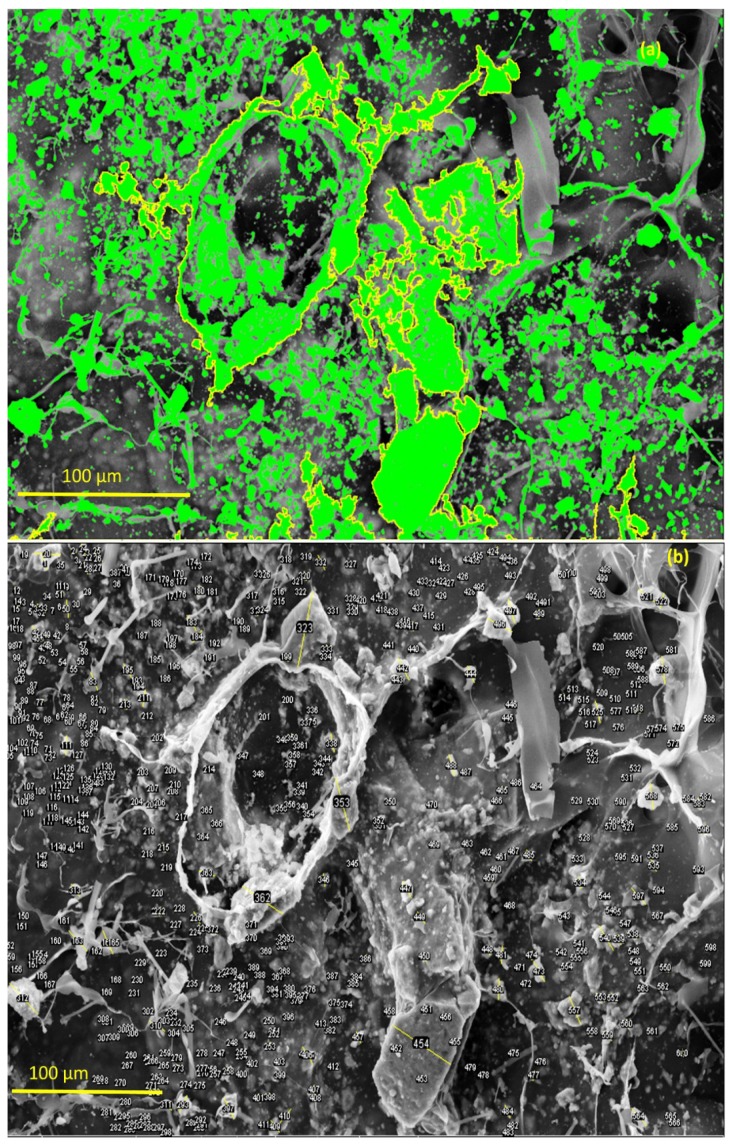
(**a**) Highlight the distribution of the Ce-HApCG particles in the composite and (**b**) selected areas that were used in counting the average size of the granules.

With the aid of ImageJ software and tool measures [59], it was possible to observe the distribution of the Ce-HApCG granules along the polymer network. The green points highlighted in Figure 10a refer to this distribution. The highlight shown by the staining allowed to observe the different size that the Ce-HAp is dispensed on the surface of the composite. It was observed that the great majority of the particles distributed were in the form of small medium-sized granules (~2 μm). However, it is possible to observe the existence of some larger granules. Figure 10b shows the labeling of the Ce-HApCG granules which was performed by ImageJ software.

**Figure 11:**
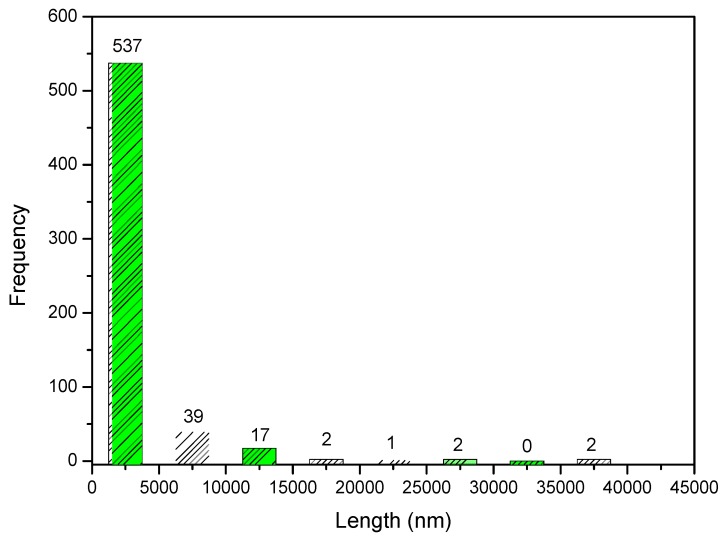
Frequency of distribution of pore size of Ce-HApCG biocomposite.

**Figure 12:**
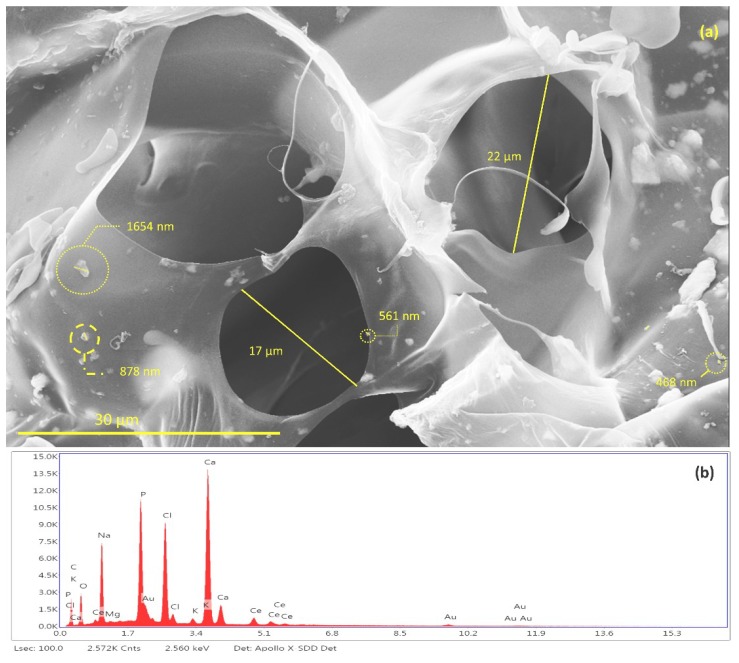
(**a**) Identification and measurement of granules of Ce-HApCG and pores of scaffolds and (**b**) energy-dispersive X-ray spectroscopy patterns of S*_HG_*.

Through 600 points selected in the micrograph, it was possible to prepare a histogram with the frequency of the size of the granules in the composite. The histogram is shown in Figure 11. From the histogram data, it can be observed that 89.5% of the measured granules have an average length of about 2.5 μm. However, when looking at Figure 12, the presence of granules at the nanoscale is noted.

It is possible to notice the existence of pores in the S*_HG_* structure (Figure 12a) with average of 19.5 ± 2.5 μm. The studies indicate a great diversity in pore size in the scaffolds synthesized with the size variation occurring in the range of 10 to 1000 μm. This variation of pore size occurs due to the different bone morphologies found in the human body, as well as in the scaffold application sites [60,61,62,63].

The energy dispersive spectroscopy (EDS) spectrum of scaffold, shown in Figure 12b, revealed the qualitative composition of the material. It was possible to identify Ca, P, O atoms from the hydroxyapatite composition. It was also possible to identify the presence of cerium that is incorporated in the HAp. Finally, the identified Na, Cl, Mg, and K atoms are derived from the GG composition. The gold identified in the EDS comes from the sample preparation process by FESEM.

Data provided by EDS relative to the atomic percentage were used to calculate the Ca/P ratio of synthesized hydroxyapatite [64,65,66]. The results show that the Ca/P ratio for Ce-HA ((Ca + Ce)/P) present in S*_HG_* was 1.87. This result is in agreement with values already reported for hydroxyapatite found in the human organism when comparing crystallinity and calcium content. The hydroxyapatite synthesized in this study does not have a high crystallinity when compared to hydroxyapatites with a Ca/P ratio = 1.67 [67]. Thus, because it does not have a high stability, it is believed that Ce-HAp has greater interaction effects with living organisms.

### 2.2. In Vitro Studies

#### 2.2.1. In Vitro Degradation Study in simulated body fluid (PBS)

**Figure 13:**
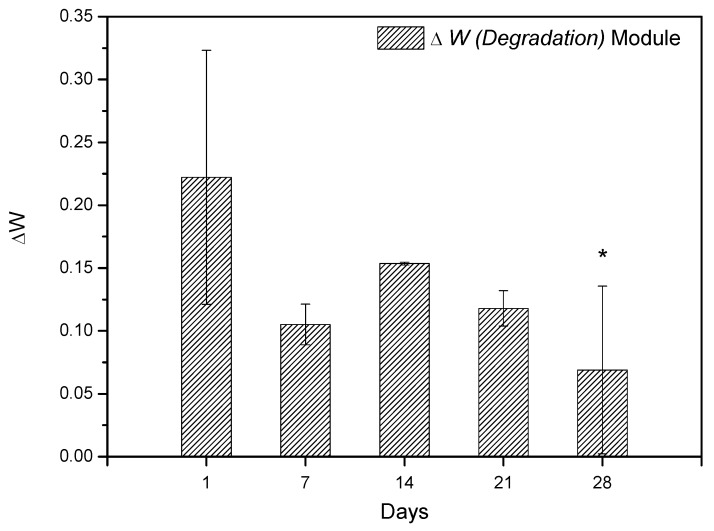
Weight variation of S*_HG_* scaffold during periods of 1, 7, 14, 21, and 28 days. The differences between the groups were analyzed by analysis of variance (ANOVA), followed by the Tukey test, comparing the groups with the value of 1 day, * represents statistically difference with 1 day.

**Figure 14:**
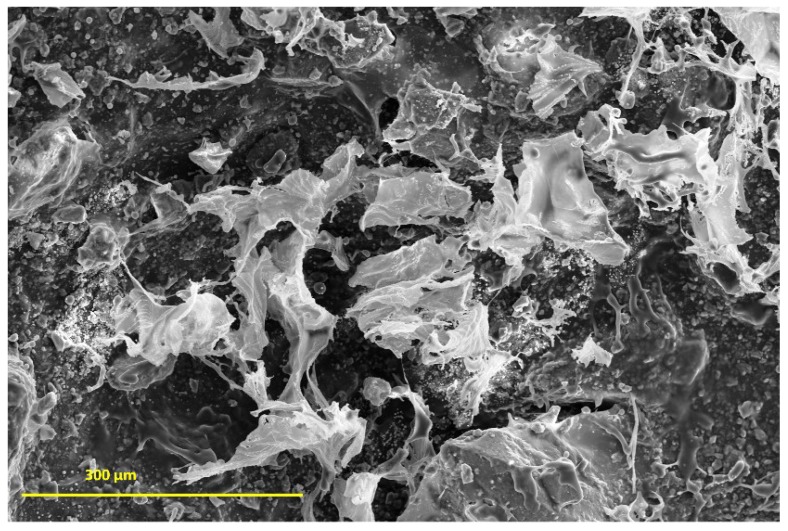
S*_HG_* SEM (Scanning Eetronic Microscopy) after 7 days immersion in PBS buffer.

In vitro degradation studies were performed by immersing the S*_HG_* in PBS. Figure 13 shows the results obtained during the degradation study. The results showed that scaffold has the ability to absorb the ions from the PBS solution in its spongy structure; thus, it is believed that the scaffold synthesized in this study may interact with body fluids [68,69] and that considering the standard deviation, a constant variation in mass is observed, regardless of immersion days in PBS. This information was obtained by comparing the values of the W*_ini_* with the W*_fin_* (Equation (1)). The amount of hydroxyapatite in the scaffold structure is fundamental for weight gain, as reported by Deb et al. [70], since the crystalline structure of the Ce-HAp can influence the scaffold not to weight loss during the degradation test, besides adding an ability to absorb the PBS in the crystal structure of the Ce-HAp [70]. Figure 14 shows the FESEM of the scaffold after soaking in PBS. It is possible to observe a thin-film incorporated on S*_HG_*, this result shows that the incorporation of Ce-HAp provides a weight increase to the biomaterial.

#### 2.2.2. MTT Assay and Hemolytic Activity

**Figure 15:**
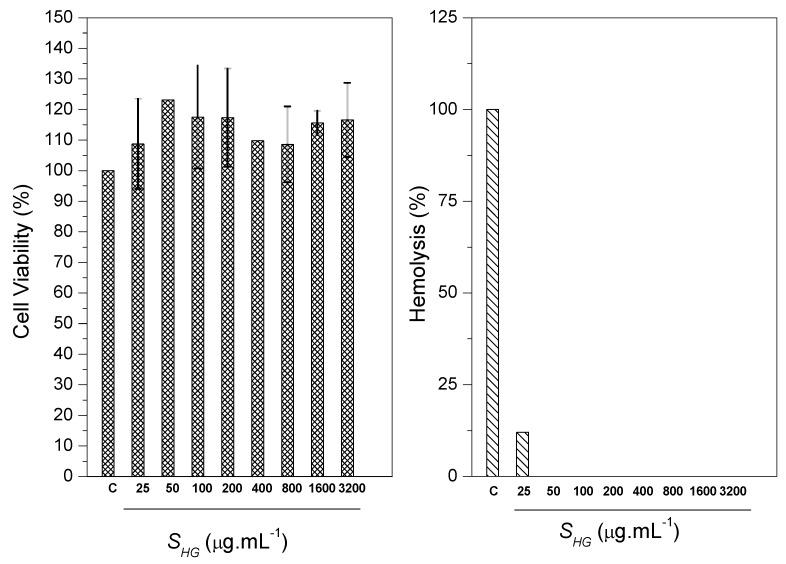
MTT assay and hemolytic activity for S*_HG_* scaffolds.

The determination of cell viability occurred by MTT assay. MTT assay was performed to observe the cytotoxicity of scaffold at serial concentrations against CTM (CC50). The positive control was given with 100% cell viability. The study showed that the number of cells cultured was significantly greater than the positive control. Among the several concentrations of scaffolds evaluated, it was noticed that none of the scaffolds presented toxic characteristics, meaning, the cell growth was not affected. The results showed that S*_HG_* scaffold presented cell growth stimulating characteristics, thus, it is believed that scaffold has a promising use in bone regeneration, since the cellular viability of the material reached values greater than 75%. The obtained values are represented as mean and standard deviation represented in the form of graphs. The results were evaluated through analysis of variance (ANOVA) with the Tukey test. The differences were considered statistically up to *p* < 0.05 (Figure 15). Therefore, the scaffold can be classified as being a non-toxic material [71,72].

The test against erythrocytes of blood of goats showed the ability of scaffold to cause hemolysis in the blood cells through lysis or cellular eruptions. Ultra pure water caused 100% hemolysis (negative control). It was observed that the scaffold presented low hemolytic action. The hemolysis induction value was 12.03%. Ramya et al. reports that according to the ASTM standard [73], materials with hemolysis <5% are considered materials that have a high level of hemocompability, and materials containing hemolysis >20% are classified as hemocompatable [74]. The results generated in this study (Figure 14) show that S*_HG_* scaffold can be classified as being hemocompatible.

### 2.3. Mechanical Testing

Being aware of the scaffold compressive strength values is important in guiding the development of the material. This property is important because it will define how the material will be applied and show the possible loads that the scaffold will support. Thus, the inverse proportionality between porosity and rigidity requires an adequate choice of which property is desired [75]. The mechanical test of the scaffold occurred by compressive strength. The S*_HG_* presented compressive strength of 19.19 MPa and modulus of elasticity of 0.24 GPa.

The obtained values classify the scaffold as being a material that has promising mechanical behavior for application as a substitute of cancellous bone, because the compressive strength found has value close to the maximum compressive strength of the cancellous bone and the modulus of elasticity found remains in the range found in the literature [73,74]. A comparison between the values obtained in this study and the values found in the literature is shown in Table 1. It is important to note that a cancellous allograft bone presents as the best option in bone surgery, being called a golden standard by specialists [60]—a finding that corroborates the results found in this study for the S*_HG_* scaffold.

## 3. Materials and Methods

### 3.1. Materials

For the development of the research, the following reagents were used: Calcium hydroxide—Ca(OH)2 (ENSURE^®^, Duque de Caxias, Rio de Janeiro, Brazil); dibasic ammonium phosphate—(NH_4_)_2_HPO_4_ (Sigma-Aldrich, St. Louis, MI, USA); and cerium nitrate—Ce(NO_3_)_3_·6H_2_O (Sigma-Aldrich); sodium hydroxide—NaOH (ISOFAR, Duque de Caxias, Rio de Janeiro, Brazil); nutrient mixture F-12 (DMEM/F-12) (Sigma-Aldrich); 3-4,5 dimethylthiazol-2,5-diphenyl tetrazolium bromide (MTT) (Sigma-Aldrich); dimethylsulfoxide (DMSO) (Mallinckrodt Chemicals, Phillipsburg, NJ, USA); cashew gum isolated; deionized water and gellan gum, kindly provided by KelcoGel^®^, Limeira, São Paulo, Brazil.

### 3.2. Cashew Gum Production

The isolation of CG occurred with the collection of the exudate present in fissures of the cashew tree (*Anacardium occidentale L.*), popularly known as cajueiro. The trees that were collected are located at the Federal University of Piauí-UFPI, SISGEN: ABD61DA. After collection, the exudate was ground by mortar and pistil, causing an initial separation between exudate and impurities. The exudate was then dissolved in distilled water in the proportion of 10.0 g per 100.0 mL of water. Dissolution of the exudate occurred by mechanical shaking for 24 h. The solution obtained was vacuum filtered to remove the remaining impurities. Next, the pH was adjusted (pH 7.0) with addition of NaOH and, finally, CG was obtained by precipitating the solution with ethanol addition. The proportion of ethanol added to the exudate solution was 3:1. After the precipitation process, the CG was centrifuged and washed with acetone three times. The gum isolation process was terminated by oven drying (24 h at 50 °C).

### 3.3. Synthesis of the Composite Based on Cerium-Doped HAp and CG

The composite synthesis was initiated by dissolving 1.0 g of the isolated CG in 20.0 mL of deionized water. After the dissolution of the CG, the synthesis of cerium-doped HAp into a CG suspension occurred. The synthesis of HAp occurred through the precipitation reaction between the precursor reagents of calcium ions (Ca^2+^—Ca(OH)_2_) and phosphate ions (PO_4_^3−^—(NH_4_)_2_HPO_4_). The doping of HAp with cerium (Ce^3+^—Ce(NO_3_)_3_·6H_2_O) occurred simultaneously in the precipitation reaction.

For the synthesis of 2.0 g of doped-hydroxyapatite containing 5% (w/w) cerium, we used 0.4342 g of Ce(NO_3_)_3_·6H_2_O), 1.4077 g of Ca(OH)_2_, and 1.5788 g of (NH_4_)_2_HPO_4_, individually dissolving each chemical reagent in about 10.0 mL of deionized water. After dissolution, the dibasic ammonium phosphate was transferred, and then the solutions of calcium hydroxide and cerium nitrate were added at the same time. The mixture was kept under stirring for 4 h at room temperature, where it remained standing for 12 h. The procedure followed with centrifugation of the solution and discarding the supernatant. The material obtained was oven dried at 100 °C for 24 h, then the composite was obtained. Finally, the composite was dried (100 °C for 24 h) and macerated with mortar and pestle. The weight ratio of Ce-HAp to CG is about 2:1. The composite was named Ce-HApCG.

### 3.4. Synthesis of Scaffolds

The production of the scaffolds started by mixing 1.0 g of composite Ce-HApCG powder with 0.2 g of GG powder. The homogenization of the mixture occurred under mechanical stirring for about 10 min in aqueous medium. A hydrogel was obtained, and then about 2000 μL of the hydrogel was placed in a 24-well plate, followed by freezing (−20 °C for 24 h). The scaffolds were obtained by the freeze-dried process for about 24 h. The scaffold based on Ce-HApCG+GG was named S*_HG_*.

### 3.5. Characterization of Materials

The characterization of Ce-HAp, composite and scaffold, occurred with the following specifications:

#### 3.5.1. Characterization of the Ce-HAp

The synthesized Ce-HAp powders were characterized by the X-ray diffraction (XRD) technique (Shimadzu (LABX-XDR 600, Shimadzu, Kyoto, Japan) with Cu-Kα (λ = 1.5406 Å)). The scanning ranges from 10 to 75° at a speed of 2° min^−1^ and exposure time of 40 min. Phase composition identification was performed by the Rietveld refinement using GSAS EXPGUI 2012 software. The scanning range for Rietveld refinement was from 10 to 110° at a speed of 1° min^−1^. The X-ray photoemission spectra (XPS) was performed with a spectrometer system (ESCA+, Scienta-Omicron) equipped with a hemispherical analyzer (EA125) and a monochromatic radiation source in Al Kα (Xm1000, 1486.7 eV). The X-ray source was used with a power of 280 W as the spectrometer worked in a constant pass energy mode of 50 eV.

#### 3.5.2. Characterization of Scaffolds

The scaffolds were analyzed by Attenuated total reflectance*/*Fourier transform infrared spectroscopy *(*ATR*/*FTIR) using a Spectrometer (Brucker Optics—Vertex 70, Brucker, Billerica, MA, USA) in a scanning range from 400–4000 cm^−1^. Thermogravimetric analysis (TGA) was performed on an SDT Q600 (V20.9 Build 20, TA Instruments, New Castle, DE, USA) instrument using 5 mg of sample with a heating rate of 10 °C min^−1^ (25 to 1000 °C), under argon atmosphere, with 100 mL min^−1^ in an alumina sample port. The morphological analysis of the materials was investigated using a field emission scanning electron microscopy (FESEM) (QUANTA 250 FEI, FEI Company, Eindhoven, The Netherlands)coupled with elemental analysis by energy dispersive spectroscopy (EDS) (EDAX Apollo X, FEI Company, Eindhoven, The Netherlands). The images were analyzed with the imageJ software (National Institures of Health, Bethesda, MD, USA), observing particle size as well as promoting the highlighting of particles by means of the change of coloration. The mechanical test was performed on scaffolds with a cylindrical area of 13.48 mm in diameter and 13.54 mm in height, at a speed of 5 mm min^−1^. The tests were performed on SHIMADZU: AG-X 250 kN servo-hydraulic equipment for mechanical testing (ASTM Standards D 2990-01).

### 3.6. In Vitro Studies

#### 3.6.1. In Vitro Degradation Studies

In vitro degradation studies were performed by immersing the scaffolds in simulated body fluid (PBS). The scaffolds were weighed on an analytical balance, and then samples were immersed in PBS at 37 °C in a drying oven. The period of the degradation studies occurred in the following intervals: 1, 7, 14, 21, and 28 days, replacing PBS each 7 days. The calculation of the degradation studies was performed by Equation (1) [76]:

(1) $$W=\frac{\left(W_{ini}-W_{fin}\right)}{W_{ini}},$$

where *W_ini_* was the initial weight of scaffolds and *W_fin_* was the final weight of scaffold after soaking.

#### 3.6.2. MTT study

Cell viability was analyzed by the MTT colorimetric assay (3-(4.5-dimethylthiazol-2yl))-2.5 diphenyl tetrazolium bromide. The S*_HG_* scaffold was diluted in dimethylsulfoxide (DMSO) to provide a stock solution (80 mg mL^−1^). In each assay, the stock solution was diluted in DMEM/F12 culture medium to the desired concentrations with a maximum concentration of DMSO of about 0.2% v/v. The cells used in the MTT assay were mesenchymal stem cells of rabbits (MSCs). The study was approved by the Ethics Committee on Animal Use (CEUA/UFPI), n° 021/14.

In the sequence, 100 μL of DMEM/F12 medium supplemented and about 2 × 103 CTM were added in 96-well plates. The plates were incubated at 37 °C in a humidified atmosphere containing 5% CO_2_ and 95% air atmospheric for 24 h. After cell adhesion occurred, washes (2×) were performed with DMEM/F12 medium supplemented to remove non-adherent cells. Subsequently, in each well, 100 μL of DMEM/F12 medium supplemented with different concentrations of the separately tested solutions (25, 50, 100, 200, 400, 800, 1600, and 3200 μg mL^−1^) were added. Then the cells were incubated for 48 h, after that time, 10 μL of MTT diluted in DMEM/F12 medium (5 mg mL^−1^) was added.

The plates were incubated in an oven at 37 °C in a humidified atmosphere containing 5% CO_2_ and 95% air atmospheric for 4 h, the supernatant was discarded and 100 μL of DMSO was added to all wells. The 96-well plates were under constant stirring (Kline agitator, model AK 0506) for about 30 min at room temperature for complete dissolution of the formazan. Optical density (OD) values obtained at a wavelength (λ = 550 nm) in a spectrophotometer were converted into percentages of cell viability relative. The procedure was performed in triplicate and the 50% cytotoxic concentration (CC50) was defined as the dose of the material required for the reduction of cell viability by 50%. The positive control group was converted in percentages in relation to the control group, considered to be 100%, and the negative control was performed with DMEM/F12 medium at 0.2% (v/v) DMSO [77].

#### 3.6.3. Hemolytic Activity

For its evaluation, hemolytic activity was performed on erythrocytes of blood of goats. The erythrocytes were collected with anticoagulant (EDTA). The study was approved by the Ethics Committee on Animal Use (CEUA/UFPI), n° 117/15. After collection, the erythrocytes were diluted in 80 μL of PBS and the blood concentration was adjusted (5% red blood cells). Next, S*_HG_* scaffold was diluted with the addition of PBS (20 μL). Immediately after, the erythrocytes were incubated (1 h at 37 °C) and the reaction was stopped to re-add of PBS (200 μL). The suspension was centrifuged (1000 G, 10 min) at room temperature. After centrifugation, the supernatant was measured at 550 nm to quantify the hemolytic activity. The absence (negative control) and 100% hemolysis (positive control) were determined, replacing the sample solution tested with equal volume of PBS and ultrapure water, respectively. The procedure was performed in triplicate and the results were expressed as percentage and hemolytic concentration (CH50) for 50% of erythrocytes considering the positive control as 100% hemolysis [78].

## 4. Conclusions

The study provided the synthesis of an innovative scaffold with promising potential for application in bone grafts obtained by combining a modified inorganic material (Ce-HAp) with non-toxic, low-cost renewable biopolymers. The structural characterizations, XRD and FTIR, show the success of obtaining Ce-HAp by chemical precipitation method, forming a composite constituted of cashew gum and Ce-HAp. The XPS shows the presence of cerium in the composite, as well as EDS, which in addition to the presence of cerium, showed the semi-quantitative ratio (Ca + Ce)/P = 1.87. Thermal analysis complemented with the identification of cashew gum and Ce-HAp in the biocomposite. Microscopies revealed the morphology of the scaffold, showing the dispersion of the nanometric granules of Ce-HApCG in the GG polymer matrix. The micrographs also showed the existence of pores, necessary in the osteoinduction process. The degradation study showed that the scaffold interacted with the PBS ions, a fact evidenced by scaffold weight gain and by the observation of a thin-film on the surface of the scaffold. The scaffold showed that it has adequate compression strength and that it has properties that can be used to replace the cancellous bone. MTT assay and hemolysis activity revealed that the material is non-toxic and is apt to be evaluated in some in vivo studies.

## Figures and Tables

**Table 1:** Comparison between compressive strength and modulus of elasticity of S*_HG_*, cancellous bone and cortical bone.

Sample	Compressive Strength (MPa)	Modulus of Elasticity (GPa)
S*_HG_*	19.19	0.24
cancellous bone [73,74]	4–12	0.1–0.5
cortical bone [73,74]	130–180	12–18

## References

[1] Elliot J.C. (1994). Structure and Chemistry of the Apatites and Other Calcium Orthophosphates.

[2] Levitt S.R., Crayton P.H., Monroe E.A., Condrate R.A. (1969). Forming method for apatite prostheses. J. Biomed. Mater. Res..

[3] Monroe E.A., Votava W.V., Bass D.B., McMullen J. (1971). New calcium phosphate ceramic material for bone. J. Dent. Res..

[4] Yilmaz B., Alshemary A.Z., Evis Z. (2019). Co-doped hydroxyapatites as potential materials for biomedical applications. Microchem. J..

[5] Shim K.S., Kim H.J., Kim S.E., Park K. (2018). Simple surface biofunctionalization of biphasic calcium phosphates for improving osteogenic activity and bone tissue regeneration. J. Ind. Eng. Chem..

[6] Li Y., Jiang T., Zheng L., Zhao J. (2017). Osteogenic differentiation of mesenchymal stem cells (MSCs) induced by three calcium phosphate ceramic (CaP) powders: A comparative study. Mater. Sci. Eng. C.

[7] Zhang J., Wu H., He F., Wu T., Zhou L., Ye J. (2019). Concentration-dependent osteogenic and angiogenic biological performances of calcium phosphate cement modified with copper ions. Mater. Sci. Eng. C.

[8] Barakat N.A.M., Khil M.S., Omran A.M., Sheikh F.A., Kim H.Y. (2009). Extraction of pure natural hydroxyapatite from the bovine bones bio waste by three different methods. J. Mater. Process. Technol..

[9] Pietak A.M., Reid J.W., Stott M.J., Sayer M. (2007). Silicon substitution in the calcium phosphate bioceramics. Biomaterials.

[10] Sygnatowicz A., Keyshar K., Tiwari A. (2010). Antimicrobial Properties of Silver-doped Hydroxyapatite Nano-powders and Thin Films. Biol. Biomed. Mater..

[11] Wong K.L., Wong C.T., Liu W.C., Pan H.B., Fong M.K., Lam W.M., Cheung W.L., Tang W.M., Chiu K.Y., Luk K.D.K. (2009). Mechanical properties and in vitro response of strontium-containing hydroxyapatite/polyetheretherketone composites. Biomaterials.

[12] Suganthi R.V., Elayaraja K., Joshy M.I.A., Chandra V.S., Girija E.K., Kalkura S.N. (2011). Fibrous growth of strontium substituted hydroxyapatite and its drug release. Mater. Sci. Eng. C.

[13] Kalita S.J., Bose S., Hosick H.L., Bandyopadhyay A. (2004). CaO-P2O5-Na2O-based sintering additives for hydroxyapatite (HAp) ceramics. Biomaterials.

[14] TWebster J., Massa-Schlueter E.A., Smith J.L., Slamovich E.B. (2004). Osteoblast response to hydroxyapatite doped with divalent and trivalent cations. Biomaterials.

[15] Joshy M.I.A., Elayaraja K., Suganthi R.V., Veerla S.C., Kalkura S.N. (2011). In vitro sustained release of amoxicillin from lanthanum hydroxyapatite nano rods. Curr. Appl. Phys..

[16] Yasukawa A., Kandori K., Tanaka H., Gotoh K. (2012). Preparation and structure of carbonated calcium hydroxyapatite substituted with heavy rare earth ions. Mater. Res. Bull..

[17] Yingguang L., Zhuoru Y., Jiang C. (2007). Preparation, Characterization and Antibacterial Property of Cerium Substituted Hydroxyapatite Nanoparticles. J. Rare Earths.

[18] Greenhalgh D.G. (2009). Topical Antimicrobial Agents for Burn Wounds. Clin. Plast. Surg..

[19] Ball J.P., Mound B.A., Monsalve A.G., Nino J.C., Allen J.B. (2015). Biocompatibility evaluation of porous ceria foams for orthopedic tissue engineering. J. Biomed. Mater. Res. Part A.

[20] Emsley J. (2011). Nature’s Building Blocks: An A-Z Guide to the Elements, New Editio.

[21] Jakupec M.A., Unfried P., Keppler B.K. (2005). Pharmacological properties of cerium compounds. Rev. Physiol. Biochem. Pharmacol..

[22] Aparecida A.H., Vinícius M., Fook L., Luis M., Guastaldi C., de Físico-química D., de Química I., Paulista U.E., Degni R.F. (2007). Estudo da influência dos iões K^+^,Mg^2+^,SO_4_^2-^ e CO_3_^2-^ na cristalização biomimétrica de fosfato de cálcio amorfo (ACP) e conversão a fofato octacálcico (OCP). Quím. Nova.

[23] Aguiar A.E., Silva M.d., Rodas A.C.D., Bertran C.A. (2019). Mineralized layered films of xanthan and chitosan stabilized by polysaccharide interactions: A promising material for bone tissue repair. Carbohydr. Polym..

[24] Liu J., Willför S., Xu C. (2015). A review of bioactive plant polysaccharides: Biological activities, functionalization, and biomedical applications. Bioact. Carbohydrates Diet. Fibre.

[25] de Paula R.C.M., Heatley F., Budd P.M. (1998). Characterization of Anacardium occidentale Exudate Polysaccharide. Polym. Int..

[26] de Paula H.C.B., de Oliveira E.F., Abreu F.O.M.S., de Paula R.C.M., de Morais S.M., Forte M.M.C. (2010). Esferas (Beads ) de Alginato como Agente Encapsulante de Óleo de Croton Zehntneri Pax et Hoffm ALG/Ca Beads as an Encapsulation Agent of Croton Zehntneri Pax et Hoffm Essential Oil. Polímeros Ciência e Tecnologia.

[27] Ribeiro A.J., de Souza F.R.L., Bezerra J.M., Oliveira C., Nadvorny D., Monica F., Nunes L.C., Silva-Filho E.C., Veiga F., Sobrinho J.L.S. (2016). Gums’ based delivery systems: Review on cashew gum and its derivatives. Carbohydr. Polym..

[28] Florêncio G.V.S.F.M., de Oliveira Silva M.F., Ana J.L.D.L.F., Leão M.D.A.C., Porto A.L.F. (2006). O polissacarídeo do Anacardium occidentale L. na fase inflamatória do processo cicatricial de lesões cutâneas. Ciência Rural.

[29] Ribeiro A.C., Eaton P., Campos D.A., Eiras C., Pintado M.M., Leite J.R.S.A., Costa E.M., Araruna F.B., Tavaria F.K., Fernandes J.C. (2012). Study of antimicrobial activity and atomic force microscopy imaging of the action mechanism of cashew tree gum. Carbohydr. Polym..

[30] Shingala V.K., Singh A.K., Yadav S.K., Sivakumar T. (2010). Design and characterization of diclofenac sodium tablets containing Mangifera indica resin as release retardant. Int. J. PharmTech Res..

[31] Ofori-Kwakye K., Asantewaa Y., Kipo S.L. (2010). Physicochemical and binding properties of cashew tree gum in metronidazole tablet formulations. Int. J. Pharm. Pharm. Sci..

[32] Da Silveira Nogueira Lima R., Rabelo Lima J., Ribeiro de Salis C., de Azevedo Moreira R. (2002). Moreira, Cashew-tree (Anacardium occidentale L.) exudate gum: A novel bioligand tool. Biotechnol. Appl. Biochem..

[33] Gyedu-Akoto E., Oduro I. (2008). Physico-chemical properties of cashew tree gum. African J. Food Sci..

[34] FAO (1972). Food and Agriculture Organization of the United Nations. Encycl. Toxicol..

[35] Augusto Lopes S.L., de Paula Pesoa P.F.A., Serrano L.A.L. (2017). Aspectos econômicos da cultura do cajueiro. Sistema de Produção do Caju.

[36] Zia K.M., Tabasum S., Khan M.F., Akram N., Akhter N., Noreen A., Zuber M. (2018). Recent trends on gellan gum blends with natural and synthetic polymers: A review. Int. J. Biol. Macromol..

[37] Jansson P.E., Lindberg B., Sandford P.A. (1983). Structural Studies of Gellan Gum, an extracellular polysaccharide elaborated by Pseudomonas elodea. Carbohydr. Res..

[38] dos Reis Corrêa L.T., de Laia A.G.S., de Souza Costa H. Processamento e caracterização de hidrogéis a base de alginato e goma gelana visando aplicações em articulações. Proceedings of the 14° Congresso da Sociedade Latino Americana de Biomateriais, Orgãos Artificiais e Engenharia de Tecidos - SLABO5ª Edição do Workshop de Biomateriais, Engenharia de Tecidos e Orgãos Artificiais—OBI20.

[39] Lorenzo G., Zaritzky N., Califano A. (2013). Rheological analysis of emulsion-filled gels based on high acyl gellan gum. Food Hydrocoll..

[40] Salarian M., Solati-Hashjin M., Shafiei S.S., Goudarzi A., Salarian R., Nemati A. (2009). Surfactant-assisted synthesis and characterization of hydroxyapatite nanorods under hydrothermal conditions. Mater. Sci..

[41] Kolodziejczak-Radzimska A., Samuel M., Paukszta D., Piasecki A., Jesionowski T. (2014). Synthesis of hydroxyapatite in the presence of anionic surfactant. Physicochem. Probl. Miner. Process..

[42] Sofronia A.M., Baies R., Anghel E.M., Marinescu C.A., Tanasescu S. (2014). Thermal and structural characterization of synthetic and natural nanocrystalline hydroxyapatite. Mater. Sci. Eng. C.

[43] Mu Y., Zhu K., Luan J., Zhang S., Zhang C., Na R., Yang Y., Zhang X., Wang G. (2019). Fabrication of hybrid ultrafiltration membranes with improved water separation properties by incorporating environmentally friendly taurine modified hydroxyapatite nanotubes. J. Memb. Sci..

[44] Sarkar C., Sahu S.K., Sinha A., Chakraborty J., Garai S., Mu Y., Zhu K., Luan J., Zhang S., Zhang C. (2019). Facile synthesis of carbon fiber reinforced polymer-hydroxyapatite ternary composite: A mechanically strong bioactive bone graft. J. Memb. Sci..

[45] Fahami A., Nasiri-Tabrizi B., Beall G.W., Basirun W.J. (2017). Structural insights of mechanically induced aluminum-doped hydroxyapatite nanoparticles by Rietveld refinement. Chin. J. Chem. Eng..

[46] Momma K., Izumi F. (2011). VESTA 3 for three-dimensional visualization of crystal, volumetric and morphology data. J. Appl. Crystallogr..

[47] Uhart A., Ledeuil J.B., Gonbeau D., Dupin J.C., Bonino J.P., Ansart F., Esteban J. (2016). An Auger and XPS survey of cerium active corrosion protection for AA2024-T3 aluminum alloy. Appl. Surf. Sci..

[48] Pavia J.R., Lampman D.L., Kriz G.M., Vyvyan G.S. (2009). Introduction to Spectroscopy.

[49] Pitombeira N.A.O., Neto J.G.V., Silva D.A., Feitosa J.P.A., Paula H.C.B., de Paula R.C.M. (2015). Self-assembled nanoparticles of acetylated cashew gum: Characterization and evaluation as potential drug carrier. Carbohydr. Polym..

[50] Quelemes P.V., de Araújo A.R., Plácido A., Delerue-Matos C., Maciel J.S., Bessa L.J., Ombredane A.S., Joanitti G.A., Soares M.J.d.S., Eaton P. (2017). Quaternized cashew gum: An anti-staphylococcal and biocompatible cationic polymer for biotechnological applications. Carbohydr. Polym..

[51] Pereira D.R., Silva-Correia J., Oliveira J.M., Reis R.L., Pandit A., Biggs M.J. (2018). Nanocellulose reinforced gellan-gum hydrogels as potential biological substitutes for annulus fibrosus tissue regeneration, Nanomedicine Nanotechnology. Biol. Med..

[52] Lee M.W., Tsai H.F., Wen S.M., Huang C.H. (2012). Photocrosslinkable gellan gum film as an anti-adhesion barrier. Carbohydr. Polym..

[53] Mothé C.G., Rao M.A. (2000). Thermal behavior of gum arabic in comparison with cashew gum. Thermochim. Acta.

[54] Sun F., Lim B.K., Ryu S.C., Lee D., Lee J. (2010). Preparation of multi-layered film of hydroxyapatite and chitosan. Mater. Sci. Eng. C.

[55] Gonsalves J.K.M.C., Ferro J.N.S., Barreto E.O., Nunes R.S., Valerio M.E.G. (2016). In fl uence of concentration of hydroxyapatite surface modi fi er agent on bioactive composite characteristics. Ceram. Int..

[56] Prezotti F.G., Stringhetti B., Cury F., Evangelista R.C. (2014). Mucoadhesive beads of gellan gum/pectin intended to controlled delivery of drugs. Carbohydr. Polym..

[57] Verma A., Nagarwal R. (2012). Preparation and Characterization of Floating Gellan-Chitosan Polyelectrolyte Preparation and Characterization of Floating Gellan-Chitosan Polyelectrolyte Complex Beads. Latin Am. J. Pharm..

[58] Leung C.H., Mak C.S.K., Pong P.W.T., Wu E.X., Leung C.W., Shi J., Chan K.Y., Wong C.M., Chan N.M.M., Mak K.Y. (2013). Sterilization on dextran-coated iron oxide nanoparticles: Effects of autoclaving, filtration, UV irradiation, and ethanol treatment. Microelectron. Eng..

[59] Schneider C.A., Rasband W.S., Eliceiri K.W. (2012). NIH Image to ImageJ: 25 years of image analysis. Nat. Methods.

[60] Chen S., Shi Y., Zhang X., Ma J. (2019). 3D printed hydroxyapatite composite scaffolds with enhanced mechanical properties. Ceram. Int..

[61] Thavornyutikarn B., Chantarapanich N., Sitthiseripratip K., Thouas G.A., Chen Q. (2014). Bone tissue engineering scaffolding: Computer-aided scaffolding techniques. Prog. Biomater..

[62] Roseti L., Parisi V., Petretta M., Cavallo C., Desando G., Bartolotti I., Grigolo B. (2017). Scaffolds for Bone Tissue Engineering: State of the art and new perspectives. Mater. Sci. Eng. C.

[63] Shavandi A., Bekhit A.E.D.A., Sun Z., Ali M.A. (2016). Bio-scaffolds produced from irradiated squid pen and crab chitosan with hydroxyapatite/β-tricalcium phosphate for bone-tissue engineering. Int. J. Biol. Macromol..

[64] Kanchana P., Navaneethan M., Sekar C. (2017). Fabrication of Ce doped hydroxyapatite nanoparticles based non-enzymatic electrochemical sensor for the simultaneous determination of norepinephrine, uric acid and tyrosine. Mater. Sci. Eng. B Solid-State Mater. Adv. Technol..

[65] Yang Y.C., Chen C.C., Wang J.B., Wang Y.C., Lin F.H. (2017). Flame sprayed zinc doped hydroxyapatite coating with antibacterial and biocompatible properties. Ceram. Int..

[66] Ren Y., Zhou H., Nabiyouni M., Bhaduri S.B. (2015). Rapid coating of AZ31 magnesium alloy with calcium deficient hydroxyapatite using microwave energy. Mater. Sci. Eng. C.

[67] Sobczak-Kupiec A., Olender E., Malina D., Tyliszczak B. (2018). Effect of calcination parameters on behavior of bone hydroxyapatite in artificial saliva and its biosafety. Mater. Chem. Phys..

[68] Okada M., Matsumoto T. (2015). Synthesis and modification of apatite nanoparticles for use in dental and medical applications. Jpn. Dent. Sci. Rev..

[69] Arifta T.I., Munar M.L., Tsuru K., Ishikawa K. (2017). Fabrication of interconnected porous calcium-deficient hydroxyapatite using the setting reaction of α tricalcium phosphate spherical granules. Ceram. Int..

[70] Deb P., Barua E., Deoghare A.B., Lala S.D. (2019). Development of bone scaffold using Puntius conchonius fish scale derived hydroxyapatite: Physico-mechanical and bioactivity evaluations. Ceram. Int..

[71] Hirani A.A., Dong S., Colacino K.R., Roman M., Lee Y.W. (2012). Cytotoxicity and Cellular Uptake of Cellulose Nanocrystals. Nano Life.

[72] Niamsap T., Lam N.T., Sukyai P. (2019). Production of hydroxyapatite-bacterial nanocellulose scaffold with assist of cellulose nanocrystals. Carbohydr. Polym..

[73] Chowdhury S.K.R., Mishra A., Pradhan B., Saha D. (2004). Wear characteristic and biocompatibility of some polymer composite acetabular cups. Wear.

[74] Ramya J.R., Arul K.T., Sathiamurthi P., Asokan K., Kalkura S.N. (2016). Novel gamma irradiated agarose-gelatin-hydroxyapatite nanocomposite scaffolds for skin tissue regeneration. Ceram. Int..

[75] Rezwan K., Chen Q.Z., Blaker J.J., Boccaccini A.R. (2006). Biodegradable and bioactive porous polymer/inorganic composite scaffolds for bone tissue engineering. Biomaterials.

[76] Hu M., Xiao F., Ke Q.F., Li Y., Chen X.D., Guo Y.P. (2019). Cerium-doped whitlockite nanohybrid scaffolds promote new bone regeneration via SMAD signaling pathway. Chem. Eng. J..

[77] Carvalho C.E.S., Sobrinho-Junior E.P.C., Brito L.M., Nicolau L.A.D., Carvalho T.P., Moura A.K.S., Rodrigues K.A.F., Carneiro S.M.P., Arcanjo D.D.R., Citó A.M.G.L. (2017). Anti-Leishmania activity of essential oil of Myracrodruon urundeuva (Engl.) Fr. All.: Composition, cytotoxity and possible mechanisms of action. Exp. Parasitol..

[78] Steindel M., Bachère E., Barracco M.A., Miletti L.C., Löfgren S.E. (2007). Trypanocidal and leishmanicidal activities of different antimicrobial peptides (AMPs) isolated from aquatic animals. Exp. Parasitol..

